# Long-Term Survival among Patients Hospitalized for COVID-19 during the First Three Epidemic Waves: An Observational Study in a Northern Italy Hospital

**DOI:** 10.3390/ijerph192215298

**Published:** 2022-11-19

**Authors:** Marinella Bertolotti, Marta Betti, Fabio Giacchero, Chiara Grasso, Genny Franceschetti, Margherita Carotenuto, Anna Odone, Guglielmo Pacileo, Daniela Ferrante, Antonio Maconi

**Affiliations:** 1 Research Training Innovation Infrastructure, Research and Innovation Department (DAIRI), Azienda Ospedaliera SS. Antonio e Biagio e Cesare Arrigo, 15121 Alessandria, Italy; 2 Medical Directorate, Azienda Ospedaliera SS. Antonio e Biagio e Cesare Arrigo, 15121 Alessandria, Italy; 3 Department of Public Health, Experimental and Forensic Medicine, University of Pavia, 27100 Pavia, Italy; 4 Local Health Authority Alessandria, 15121 Alessandria, Italy; 5 Unit of Medical Statistics, Department of Translational Medicine, Università del Piemonte Orientale and Cancer Epidemiology Unit, CPO-Piemonte, 28100 Novara, Italy

**Keywords:** COVID-19, survival, waves, hospitalization, prognostic factors, follow-up

## Abstract

The mortality rate of hospitalized COVID-19 patients differed strongly between the first three pandemic waves. Nevertheless, their long-term survival has been poorly assessed. The aim of this study was to compare the clinical characteristics and mortality rates of 825 patients with coronavirus disease 2019 (COVID-19) infection who were hospitalized at the Alessandria hub hospital, in Northern Italy, during the first fifty days of the first three pandemic waves. Each subject was followed in terms of vital status for six months from the date of hospital admission or until deceased. Patients admitted during the three waves differed in age (*p* = 0.03), disease severity (*p* < 0.0001), Charlson comorbidity index (*p* = 0.0002), oxygen therapy (*p* = 0.002), and invasive mechanical ventilation (*p* < 0.0001). By the end of follow-up, 309 deaths (38.7%) were observed, of which 186 occurred during hub hospitalization (22.5%). Deaths were distributed differently among the waves (*p* < 0.0001), resulting in being higher amongst those subjects admitted during the first wave. The COVID-19 infection was reported as the main cause of death and patients with a higher mortality risk were those aged ≥65 years [adjusted HR = 3.40 (95% CI 2.20–5.24)], with a higher disease severity [adjusted HR = 1.87 (95%CI 1.43–2.45)], and those requiring oxygen therapy [adjusted HR = 2.30 (95%CI 1.61–3.30)]. In conclusion, COVID-19 patients admitted to our hub hospital during the second and the third waves had a lower risk of long-term mortality than those admitted during the first. Older age, more severe disease, and the need for oxygen therapy were among the strongest risk factors for poor prognosis.

## 1. Introduction

In late 2019, an acute respiratory disease was reported in Wuhan, China, caused by the severe acute respiratory syndrome coronavirus 2, also known as coronavirus disease 2019 (COVID-19). The COVID-19 infection spread rapidly worldwide, becoming a global public health emergency: it was estimated that in the first two years of the pandemic, it infected at least 440 million people and caused approximately 5.97 million deaths, with these estimates still growing rapidly [1].

The clinical manifestations of COVID-19 infection are extremely variable, ranging from a mild flu-like illness to severe pneumonia [2]. When symptomatic, the COVID-19 infection may begin with fever, cough, myalgia, headache, extreme fatigue, and dyspnea. As the disease progresses, the patient may experience symptoms such as lung inflammation, fibrosis, and edema, which gradually develop into an acute respiratory distress syndrome and, in most severe cases, lung injury, systemic inflammatory response, and extra-pulmonary multi-organ failure [3]. 

There are multiple clinical predictors of COVID-19 disease exacerbation and mortality including age and concurrent diseases: there is multiple evidence of a more life-threatening infection in older patients, especially those over 65 years of age, and in patients with comorbidities such as cardiovascular and chronic respiratory diseases, diabetes, hypertension, obesity, and cancer [4]. Some laboratory parameters including leucocyte count, AST, PCR, LDH, and D-dimer have been linked to the prognosis of COVID-19 [4,5].

Italy was the first European country to experience widespread population outreach, with the highest number of reported COVID-19 cases and high mortality rates in spring 2020. The first epidemic wave was observed between the end of February and May 2020, with a peak in March and April, but the strict and enforced lockdown from March to May massively reduced the spread of the virus, scaling down the first wave of the infection [6,7]. The second wave began in late summer of the same year, and came to a peak in late October 2020 [6].

A considerable geographical heterogeneity was reported between the first two waves in Italy: during the first epidemic period, the Northern regions were the most severely affected by the infection, whereas the Center and the Southern regions experienced a lower-impact outbreak. During the second wave, on the other hand, the circulation of the virus was more homogeneous across the country [7]. Recently, the evolution of the COVID-19 epidemic has led to further infection peaks: in Italy, the third peak occurred around 15 March 2021, with approximately 22,000 new cases diagnosed daily, while the fourth occurred later that year [8]. The third wave was dominated by a marked increase of new COVID-19 infections among young patients, partly attributed to the spread of new virus variants [9]. 

In Italy, a significant increase in mortality rates was reported during the COVID-19 pandemic [10], with an estimated mortality of 7.2%, substantially higher than that observed in China (2.3%). Notably, higher mortality rates were observed among individuals aged 70 years or older and among men [11]. 

According to the recent National Institute of Statistics (ISTAT) report, among all of the COVID-19 deaths that occurred in Italy during the first wave, 85.2% were concentrated in Northern regions, while during the second wave, within those regions, the mortality dropped to 59.4%. First- and second-wave COVID-19 mortalities in Piedmont accounted for 11.8% and 7.1% of total fatalities, respectively (source: www.istat.it, BES report 2020, accessed on 10 march 2021). 

Over time, with the protraction of the pandemic in Italy, changes in COVID-19 related deaths have been reported, and the mortality rate among COVID-19 patients hospitalized during the third wave of the epidemic in Italy were significantly lower than during the first and the second waves [8]. Studies aimed at investigating cause-specific mortality highlighted that, especially in the first period of the pandemic, the COVID-19-related diseases were the leading cause of death among men and the second cause among women, and that flu and pneumonia showed the highest increase in mortality rates [12]. 

In this study, we aimed to evaluate and compare the epidemiological and clinical characteristics as well as the mortality rates of COVID-19 infected patients during the first three COVID-19 waves that were admitted to the SS Antonio e Biagio e Cesare Arrigo hospital in Alessandria, Northwestern Italy, the hub hospital of one of the provinces most severely infected by the COVID-19 epidemic [13].

## 2. Materials and Methods

### 2.1. Study Design and Population

The present study was developed within the COVID-19 Registry, a multicenter ambispective observational study conducted at the “SS Antonio e Biagio e Cesare Arrigo” hospital of Alessandria in the Piedmont Region, Northern Italy, whose details have been described in a previous paper [5,13,14]. Briefly, all consecutive adult patients aged between 18 and 65 years admitted to the Alessandria hospital with laboratory-confirmed COVID-19 were enrolled. Specifically, COVID-19 nucleic acid was detected by nasopharyngeal swab specimens in all patients by real-time reverse-transcriptase polymerase chain reaction (RT-PCR), according to the WHO laboratory guidelines [15]. Patients discharged from the Emergency Department were excluded. The data of the admitted patients were extracted from the electronic medical records system (TrackCare) and paper-based medical records. 

The population involved in the present study consisted of subjects with COVID-19 infection who were admitted to Alessandria’s hub hospital within the first 50 days of each of the first three waves. In order to deem a starting date for the first, second, and third waves, we considered the following dates: 24 February 2020 (i.e., the first day of data availability from the Ministry of Health) for the first wave; 14 September 2020 (i.e., the first day of the 2020/21 school year) for the second wave; and 15 February 2021 (i.e., the peak of cases detected by the Italian National Institute of Health) for the third [16]. The variables extracted from the COVID-19 registry and used for the present study were demographic and clinical characteristics such as age and gender, comorbidities, COVID-19 complications, treatments administered during hospitalization, date of hospital admission and discharge, and type of discharge. The Charlson Comorbidity Index (CCI) was calculated for each patient as previously described [17], while disease severity was classified as mild/moderate and severe/critical according to the WHO interim guidance [18], following the same criteria for the classification of disease severity described in our previous publication [5].

For each of the enrolled subject, all variables included in the Registry were recorded and stored on an electronic case report form created ad hoc using the Research Electronic Data Capture platform, which is available free of charge [19]. All data were pseudonymized according to the clinical study and data protection regulations.

These patients were subjected to follow-up to check their vital status for six months after the date of hospital admission due to COVID-19 infection. The Registrar’s Offices of the town of residence were accessed to obtain the information on vital status. The causes of death, provided by the Local Health Authority Registries of Causes of Death, were coded according to the International Classification of Diseases (9th Revision). 

### 2.2. Statistical Analysis

Quantitative data were presented as the median and interquartile range (IQR). Categorical variables were summarized as scores and percentages. Associations between categorical variables were tested with Pearson chi-square test and quantitative variables between the three waves were compared using the Kruskal–Wallis test. We reported overall survival at 6 months according to the Kaplan–Meier method. Survival intervals were measured from hospital admission for COVID-19 until death or last follow-up.

We used univariable and multivariable Cox proportional hazard models to investigate the impact of the factors on the risk of death. The proportionality of hazard assumption was tested by visual inspection of the scaled Schoenfeld residual plot and by the Grambsch and Therneau non-proportionality test. Cox regression analysis was presented as hazard ratios (HRs) and 95% confidence intervals (95%CIs). A 2-sided *p*-value < 0.05 was considered statistically significant. Analyses were performed using the MedCalc software, version 20 (MedCalc Software Ltd., Ostend, Belgium) and STATA software, version 17 (StataCorp LLC, College Station, TX, USA).

## 3. Results

### 3.1. Demographic and Clinicopathological Differences among COVID-19 Patients Hospitalized during the First Three Waves

Among all the 1651 COVID-19-positive patients recruited in the COVID-19 Registry, 825 subjects (50%) who were admitted to the Alessandria COVID-19 hub hospital during the first fifty days of the first three pandemic waves were included in this study. 

The characteristics of the subjects are reported in Table 1, both in an aggregated form and for each of the three waves considered. Of the 825 patients, 464 (56.2%) were admitted during the first wave, 255 (30.9%) during the second, and 106 (12.9%) during the third.

Most patients were male (60.7%) (Table 1). The gender distribution of the hospitalized patients did not differ between the three periods (*p* = 0.74), and patients aged ≥65 years were more strongly represented in the first wave than in the others (*p* = 0.03). The median age of all those admitted was 72 years [IQR 59–81] and differed among waves (*p* = 0.01).

Patients admitted during the three waves did not differ in the number of comorbidities (*p* = 0.43) and COVID-19 complications (*p* = 0.45), while the median duration of hospitalization, CCI, and disease severity strongly differed among waves (*p* < 0.0001, *p* = 0.0002 and *p* < 0.0001, respectively) (Table 1). 

Almost all patients (N = 785; 95.2%) underwent at least one pharmacological treatment for COVID-19 infection. no differences were observed between waves. A different proportion of patients who required immediate oxygen support, and those who needed invasive mechanical ventilation was observed between waves (*p* = 0.002 and *p* < 0.0001, respectively), the proportion being higher in the first wave (Table 1).

### 3.2. Survival Analysis of COVID-19 Patients Hospitalized during the First Three Waves

Among the patients admitted to the Alessandria hub hospital during the time intervals considered, 309 deaths (38.7%) were observed up to six months after the first day of hospital admission (Table 2). There were no losses to follow up. Mortality rates differed between waves (*p* < 0.0001): the number of deaths at the end of follow-up among hospitalized patients of the first, second, and third waves were 218 (47% of patients admitted in the same period), 69 (27%), and 22 (20.7%), respectively. 

A total of 186 (22.5%) deaths occurred during hub hospital hospitalization, distributed as follows: 132 among patients admitted during the first wave (28.5%), 42 during the second (16.5%), and 12 (11.3%) during the third (*p* < 0.0001) (Table 2). A total of 39.8% of the deaths (N = 123) occurred after hospital discharge; among these, only 18 patients (14.6%) were discharged at home, while the others (N = 105; 85.4%) were transferred to spoke hospitals or other medical clinics and facilities. Among these 105 patients, 86 were transferred during the first wave (91.9%), 27 during the second (70.4%), and 10 during the third (70%). 

Median age at the time of death was 81 years (IQR 74–86); the age of patients at the time of death differed (*p* = 0.02), being higher in the second wave (84 years; IQR 77–89) (Table 2).

The most frequent causes of death (i.e., pneumonia and distress respiratory syndrome) were together responsible for 80% of the total deaths; these were both COVID-19-correlated and were distributed differently between waves (*p* = 0.006) (Table 2).

**Figure 1:**
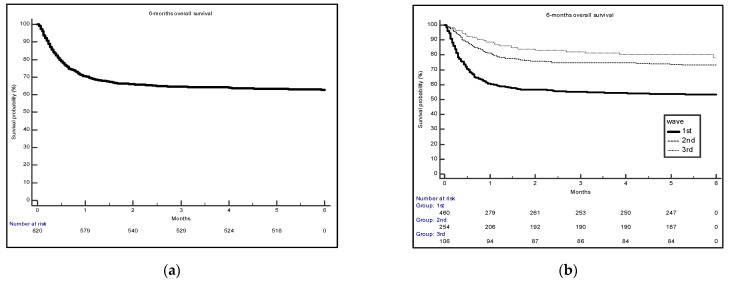
Survival probability overall (**a**) and by each of the first three pandemic waves (**b**).

Figure 1 shows the time-dependent survival probability, both overall (Figure 1a) and separately for each of the three pandemic waves (Figure 1b). The probability of mortality was lower among COVID-19 patients admitted to the hub hospital during the third wave compared to the first and second. For all waves, almost all deaths occurred within two months from hospital admission (Figure 1b).

Table 3 reports the results of the univariable and the multivariable analysis on the effect of selected characteristics in relation to the risk of death. Patients admitted during the second and the third wave had a lower risk of death within six months from the date of hospitalization in comparison with those admitted during the first (adjusted HR = 0.65; 95%CI 0.48–0.88, and adjusted HR = 0.44; 95%CI 0.28–0.70, respectively).

The multivariable analysis also showed that age (adjusted HR = 3.40; 95%CI 2.20–5.24), CCI (adjusted HR = 1.21; 95%CI 1.15–1.27), disease severity (adjusted HR = 1.87, 95%CI 1.43–2.45), and the need for oxygen therapy (adjusted HR = 2.30; 95%CI 1.61–3.30) were associated with an increased risk of death (Table 3).

## 4. Discussion

In this study, we evaluated the clinical and epidemiological characteristics and mortality risk among COVID-19 positive patients admitted to a large hub hospital in Northern Italy during the first fifty days of the first three COVID-19 pandemic waves. A total of 825 in-patients were involved, with an over-representation of men, as already reported [8,20]. The median age of the patients was 72 years, which was higher than that reported by other Italian and international studies [8,21,22,23]. More than half of the subjects were hospitalized during the first wave, while those admitted during the second and even more in the third waves were significantly fewer, in agreement with other literature [23,24,25]. In fact, although in Italy the second and third waves were more extensive than the first in terms of virus spread and number of infections, this, however, has not been paralleled by an increase in the number of hospital admissions.

Comparing the characteristics of patients hospitalized during the three waves, we found that subjects of the second wave were younger, with lower CCI, lower number of comorbidities, and milder COVID-19 disease severity; the latter feature also corresponded to a lower need of oxygen therapy. Patients admitted during the first wave were the oldest, with more severe COVID-19 disease and therefore the most in need of oxygen therapy and invasive mechanical ventilation. Moreover, they were characterized by less COVID-19-related complications and a shorter hospital in-patient period, both features presumably due to the higher mortality rates. 

Overall, we found that among the 825 patients, 38.7% died within six months from the date of admission. Mortality trends strongly differed among the waves, with a bias toward the first wave, and in most of the events, the reported cause of death was directly related to the COVID-19 infection. Indeed, 83.5% of the subjects in the first wave, 78.2% in the second, and 63.6% in the third died from COVID-19 pneumonia and distress respiratory syndrome (Table 2). 

We also evaluated the association between mortality risk and several selected clinical and epidemiological features of our patients such as gender, age, number of comorbidities, disease severity, CCI, oxygen therapy, and mechanical ventilation: all trends of the estimates were comparable with those previously reported in the literature [8,23,26].

A literature search was carried out to retrieve other studies conducted in Italy, which, like us, carried out mortality analysis for COVID-19 patients admitted to hospital during the first three pandemic waves, both to compare our results with theirs and to understand whether other research groups conducted the follow-up of patients after discharge and for how long. In general, we found that the mortality trends that we observed among waves was very similar compared to that described by many other Italian research groups [6,7,8,23,25,27,28,29], with the highest mortality rate in hospitalized patients recorded during the first wave of the pandemic followed by a gradual decrease during the subsequent peaks of infection. Almost all studies involved patients who had been admitted to hospitals in Northern Italy.

Furthermore, in our study we found that most of the death events occurred mainly within two months of hospital admission, and nearly 40% after hospital discharge. Therefore, following hospitalized COVID-19 patients after discharge, even for a few months, appears to be very important in order to not underestimate the mortality. To our knowledge, only one study prior to ours followed the patients admitted to a Northern Italian hospital during the first three waves for a few months after the admission date and then evaluated their mortality. This study, conducted on 2023 consecutive patients admitted to a COVID-19 referral center in Milan during periods similar to ours, found that 21.3%, 23.7%, and 15.8% of patients admitted during the first, second, and third waves, respectively, died within three months of admission [8]. 

Most of the studies above-mentioned considered a much shorter follow-up. Caramello and colleagues found that the mortality distribution of COVID-19 patients admitted to hospital across the whole Piedmont region in Northern Italy and followed-up for 30 days after positive COVID-19 testing was equal to 29.5%, 25%, and 19.2%, respectively, for the first, second, and third waves [25]. Two other studies also opted for a 30-day follow-up: one of these only presented the total frequency of deaths among the hospitalized patients during the three pandemic waves being equal to 20.6%, but it clearly showed the differences in the survival of subjects admitted during the first wave in the Kaplan–Meyer curves compared to the others [28]. The second study found a proportion of deaths equal to 33%, 17%, and 10%, respectively, for the first, second, and third wave [23]. Interestingly, in this latest study, the first 5 weeks of each wave were considered as the observation period, a time window that was very similar to ours [23]. Meschiari and colleagues adopted a slightly different follow-up: they reported the cumulative risk of death due to COVID-19-associated pneumonia by day 28 from admission in a tertiary care University Hospital in Northern Italy as equal to 20% for the first wave and 14.2% for the second wave [29]. 

Finally, one study did not perform any follow-up of patients after discharge, but it only considered death events that occurred during hospitalization by reporting an in-hospital mortality rate equal to 24% for the first wave, and 11% for the second/third [27]. 

To our knowledge, only one study conducted on patients hospitalized in an Italian center during the first two waves revealed an overall estimated 4-week mortality, which was higher for the second wave compared to the first, even if this difference resulted in not being significant [24].

The difference in the mortality rate observed between other Italian studies and ours in relation to the first wave could be that our center was a medical hub since the very first phase of the COVID-19 outbreak and only critically ill COVID-19 patients were referred to our hub hospital due to their need for more intensive care, while milder cases were taken to other spoke centers. Therefore, for purely organizational reasons, the centralization of patients with particularly severe disease or in need of intensive care was carried out at our hub hospital. Additionally, for logistical and organizational reasons, the hospitalized patients, once the diagnostic and therapeutic processes were completed or once the acute phase of the disease had ended, were transferred to centers with lower-intensity assistance. This necessarily first led to an over-representation in our cohort of critical patients at a higher mortality risk, also evident from the fact that the vast majority of those who died after discharge from the hub hospital were not discharged home, but transferred to other non-intensive care centers because of their need for further hospital care. In addition, it appears that our patients were older, with more comorbidities and more in need of oxygen therapy and mechanical ventilation compared to other Italian studies [8,28], which was also a consequence of the selection made by hospitalization. 

The gradual decline in the mortality rates observed from the first wave to the subsequent pandemic peaks was attributed to multiple reasons. First, the advancement in knowledge about the clinical presentation of the COVID-19 infection, but also the availability of new treatment options, improved medical care management, new radiological findings and prognostic risk factors, and improved collaboration between hospital units and preparedness of health care services, which ultimately resulted in higher patient survival [25,28,30]. 

In addition to these variables, it has been hypothesized that the characteristics of patients who died from COVID-19 infection seem to have changed after the first epidemic wave. In fact, despite the wider diffusion of the infection during the second wave, clinical and radiological evidence in hospitalized patients suggested that the severity of COVID-19-related diseases decreased after the first wave [31], as also reported in our study. Possible explanations for this observation could be the younger age and the fewer comorbidities of patients hospitalized during the second wave compared to the first, as it resulted in our cohort, but also by the diffusion of different COVID-19 variants having a higher transmissibility but a lower lethal potential [32,33], although the latter in Italy has yet to be confirmed. In contrast, it is unlikely that the COVID-19 vaccination could have contributed to reduce mortality, as the Italian vaccination campaign began in early 2021 and there was only a partial overlap between the start of the campaign and the fifty-day period of the third wave considered in our study. 

Among the strengths of our study is the availability of the cause of death and a rather long follow-up of hospitalized subjects, which allowed us not to underestimate the mortality risk for COVID-19 infection and to better characterize the long-term sequelae of COVID-19 infection in hospitalized patients. Furthermore, the collection of patient data during three consecutive pandemic waves allowed us to conduct a longitudinal assessment of changes in the clinicopathological characteristics and mortality rates of subjects requiring intensive care and hospitalized in the same hub hospital from the earliest days of the COVID-19 pandemic onward. 

This study also had limitations. The selection of subjects admitted to our hub hospital based on disease severity produced mortality estimates that differed slightly from those of other studies. Indeed, the proportion of patients who died among those hospitalized during the first wave at our center appears to be disproportionate to that of other centers in Northern Italy, which also admitted less severe cases. The addition of data from other spoke medical centers in the province that admitted patients with milder disease would allow us to have a more realistic representation of the population who has been admitted due to COVID-19 infection. 

Second, in our analysis, we arbitrary considered only admissions within the first fifty days of each wave, in contrast to other Italian studies that extended these time windows even by several months [6,8,25,27]. Therefore, we cannot exclude that this narrow selection of the observation periods may have contributed to some extent to the different estimates observed between other medical centers in Northern Italy and ours. Extending the three observation periods would certainly be useful in recovering new clues on the mortality trend among patients admitted to our hub center. 

## 5. Conclusions

As a result, we found that COVID-19 patients who were hospitalized at our hub center during the first fifty days of the first three pandemic waves significantly differed regarding both the demographic and clinicopathological characteristics. Moreover, patients admitted during the first wave resulted in being at highest risk of death compared to those hospitalized during the second and the third, with older age, worse disease, and need for oxygen therapy being among the strongest risk factors for poor prognosis. These results are substantially in line with the previously reported findings. 

What our study adds is definitely related to the rather long follow-up period, which allowed us to detect long-term deaths. Moreover, we also researched the main cause of death in order to distinguish, among all of the deaths observed in our patient series, only those directly related to COVID-19 infection.

Further retrospective evaluations of subjects with less severe COVID-19-related illness who were admitted to other spoke medical centers during the epidemic would allow us to carry out a more realistic assessment of the mortality rates of all the COVID-19 patients who had been hospitalized, regardless of the degree of disease. Moreover, by expanding the time windows of each wave, it would be possible to have an estimate of the frequency of deaths among hospitalizations in our center during each wave that is more comparable than that of other centers in Northern Italy.

## Figures and Tables

**Table 1:** Demographic and clinicopathological characteristics of the patients hospitalized at Alessandria’s hub hospital during the first fifty days of the three COVID-19 pandemic waves.

Characteristics	Total N = 825	1th Wave (24 February–13 April 2020) N = 464	2nd Wave (14 September–2 November 2020) N = 255	3rd Wave (15 February–6 April 2021) N = 106	*p*-Value
**Sex, N (%)**					
Male	501 (60.7)	287 (61.9)	152 (59.6)	62 (58.5)	0.74
Female	324 (39.3)	177 (38.1)	103 (40.4)	44 (41.5)	
**Age, N (%)**					
<65	291 (35.3)	146 (31.5)	105 (41.2)	40 (37.7)	0.03
≥65	534 (64.7)	318 (68.5)	150 (58.8)	66 (62.3)	
**Comorbidities, N (%)**					
0–1	336 (40.7)	184 (39.7)	112 (43.9)	40 (37.7)	0.43
≥2	489 (59.3)	280 (60.3)	143 (56.1)	66 (62.3)	
**Duration of hospitalization (days), median (IQR) ^1^**	11 (5–21)	9 (4–18)	14 (6–24)	17 (10–26)	0.0001
**COVID-19 complications,** **N (%)**					
No	252 (30.5)	148 (31.9)	72 (28.2)	32 (30.2)	0.45
Yes	546 (66.2)	295 (63.6)	177 (69.4)	74 (69.8)	
Missing	27 (3.3)	21 (4.5)	6 (2.4)	0	
**Disease severity, N (%)**					<0.0001
Mild/moderate	349 (42.3)	161 (34.7)	140 (54.9)	48 (45.3)	
Severe/critical	358 (43.4)	225 (48.5)	86 (33.7)	47 (44.3)	
Missing	118 (14.3)	78 (16.8)	29 (11.4)	11 (10.4)	
**Charlson Comorbidity Index, median (IQR)** ^1^	4 (2–5)	4 (3–6)	3 (2–5)	4 (2–5)	0.0002
Oxygen therapy, N (%)					
No	179 (21.7)	84 (18.1)	73 (28.6)	22 (20.8)	0.002
Yes	640 (77.6)	380 (81.9)	176 (69.0)	84 (79.2)	
Missing	6 (0.7)	0	6 (2.4)	0	
**Invasive Mechanical ventilation, N (%)**					
No	722 (87.5)	387 (83.4)	236 (92.5)	99 (93.4)	<0.0001
Yes	97 (11.8)	77 (16.6)	13 (5.1)	7 (6.6)	
Missing	6 (0.7)	0	6 (2.4)	0	

^1^ IQR: interquartile range.

**Table 2:** Description of the mortality of patients hospitalized during the first fifty days of the first three COVID-19 pandemic waves.

Characteristics	Total N = 825	1th Wave (24 February–13 April 2020) N = 464	2nd Wave (14 September–2 November 2020) N = 255	3rd Wave (15 February–6 April 2021) N = 106	*p*-Value
**Death events by the end of follow-up, N (%)**	309 (37.5)	218 (47.0)	69 (27.0)	22 (20.7)	<0.0001
**Death events during hospitalization at the Alessandria’s hub hospital, N (%)**	186 (22.5)	132 (28.5)	42 (16.5)	12 (11.3)	<0.0001
**Age at death (years), median (IQR) ^1^**	81 (74–86)	80 (73–85)	84 (77–89)	78 (74–83)	0.02
**Cause of death N (%)**					
COVID-19 pneumonia	190 (61.5)	133 (61.0)	47 (68.1)	10 (45.4)	0.006
Distress respiratory syndrome	60 (19.4)	49 (22.5)	7 (10.1)	4 (18.2)	
Other	43 (13.9)	24 (5.2)	11 (15.9)	8 (36.4)	
Missing	16 (5.2)	12 (2.6)	4 (5.8)	0	

^1^ IQR: interquartile range.

**Table 3:** Cox regression models for the risk of death at 6 months.

Characteristics	N. Deaths ^1^	Person-Months ^1^	Univariable Model	Multivariable Model ^2^
			HR ^3^ (95% CI)	HR ^3^ (95% CI)
**Wave**				
1st	172	1382	1	1
2nd	60	1019	0.48 (0.36–0.63)	0.65 (0.48–0.88)
3rd	21	460	0.35 (0.23–0.55)	0.44 (0.28–0.70)
**Sex**				
Male	152	1719	1	1
Female	101	1143	0.88 (0.70–1.12)	1.03 (0.80–1.34)
**Age**				
<65	26	1347	1	1
≥5	227	1515	6.87 (4.71–10.02)	3.40 (2.20–5.24)
**Comorbidities**				
0–1	52	1407	1	1
≥2	201	1455	3.31 (2.50–4.39)	1.28 (0.91–1.80)
**Disease severity**				
Mild/moderate	82	1652	1	1
Severe/critical	171	1210	2.46 (1.90–3.20)	1.87 (1.43–2.45)
**Charlson Comorbidity Index**	253	2862	1.26 (1.22–1.31)	1.21 (1.15–1.27)
**Oxygen therapy**				
No	40	632	1	1
Yes	213	2230	1.75 (1.27–2.39)	2.30 (1.61–3.30)
**Invasive Mechanical ventilation**				
No	208	2626	1	1
Yes	45	236	1.93 (1.44–2.58)	1.16 (0.82–1.65)

^1^ Number of deaths and person-months are reported for the subjects included in the multivariable analysis. ^2^ Each mutually adjusted for the other variables. ^3^ HR: hazard ratio.

## Data Availability

The datasets analyzed during the current study are available upon reasonable request from the corresponding author.
